# Mildly Reduced Doses of Adrenaline Do Not Affect Key Hemodynamic Parameters during Cardio-Pulmonary Resuscitation in a Pig Model of Cardiac Arrest

**DOI:** 10.3390/jcm10204674

**Published:** 2021-10-12

**Authors:** Deborah Jaeger, Jonathan Koger, Helene Duhem, Caroline Fritz, Victor Jeangeorges, Kevin Duarte, Bruno Levy, Guillaume Debaty, Tahar Chouihed

**Affiliations:** 1 SAMU-SMUR, Service d’Urgences, CHRU Nancy, 54000 Nancy, France; deborahjaeger@yahoo.com (D.J.); koger.jonathan@free.fr (J.K.); victor.jeangeorges@gmail.com (V.J.); 2 INSERM, Université de Lorraine, 54000 Nancy, France; fritzcaro@gmail.com (C.F.); blevy5463@gmail.com (B.L.); 3 Service d’Urgences, Université de Grenoble Alpes/CNRS/CHU de Grenoble Alpes, 38000 Grenoble, France; HDuhem@chu-grenoble.fr (H.D.); GDebaty@chu-grenoble.fr (G.D.); 4 Département d’Anesthésie et de Réanimation, HEGP, Assistance Publique–Hôpitaux de Paris, 75015 Paris, France; 5 Centre d’Investigation Clinique Plurithématique, INSERM, Université de Lorraine, 54000 Nancy, France; K.DUARTE@chru-nancy.fr; 6 Service de Réanimation Médicale Brabois, Pôle Cardio-Médico-Chirurgical, CHRU Nancy, 54000 Nancy, France

**Keywords:** cardiac arrest, adrenaline, coronary perfusion pressure, resuscitation

## Abstract

Adrenaline is recommended for cardiac arrest resuscitation, but its effectiveness has been questioned recently. Achieving return of spontaneous circulation (ROSC) is essential and is obtained by increasing coronary perfusion pressure (CPP) after adrenaline injection. A threshold as high as 35 mmHg of CPP may be necessary to obtain ROSC, but increasing doses of adrenaline might be harmful to the brain. Our study aimed to compare the increase in CPP with reduced doses of adrenaline to the recommended 1 mg dose in a pig model of cardiac arrest. Fifteen domestic pigs were randomized into three groups according to the adrenaline doses: 1 mg, 0.5 mg, or 0.25 mg administered every 5 min. Cardiac arrest was induced by ventricular fibrillation; after 5 min of no-flow, mechanical chest compression was resumed. The Wilcoxon test and Kruskal–Wallis exact test were used for the comparison of groups. Fisher’s exact test was used to compare categorical variables. CPP, EtCO_2_ level, cerebral, and tissue near-infrared spectroscopy (NIRS) were measured. CPP was significantly lower in the 0.25 mg group 90 s after the first adrenaline injection: 28.9 (21.2; 35.4) vs. 53.8 (37.8; 58.2) in the 1 mg group (*p* = 0.008), while there was no significant difference with 0.5 mg 39.6 (32.7; 52.5) (*p* = 0.056). Overall, 0.25 mg did not achieve the threshold of 35 mmHg. EtCO_2_ levels were higher at T12 and T14 in the 0.5 mg than in the standard group: 32 (23; 35) vs. 19 (16; 26) and 26 (20; 34) vs. 19 (12; 22) (*p* < 0.05). Cerebral and tissue NIRS did not show a significant difference between the three groups. CPP after 0.5 mg boluses of adrenaline was not significantly different from the recommended 1 mg in our model of cardiac arrest.

## 1. Introduction

Cardiac arrest is a leading cause of death and neurological impairment. Survival rates are low—around 8% in Europe—and over 50,000 people die of cardiac arrest every year in France [1,2]. In addition, the risk of severe neurological impairment is a serious public health issue, although the exact cost remains unknown [3].

The European Resuscitation Council recommends administering 1 mg of adrenaline every 3 to 5 min during cardio-pulmonary resuscitation (CPR) [4]. However, although it has been used for decades, the use of adrenaline in cardiac arrest has been questioned recently [5]. Randomized controlled trials have compared adrenaline to a placebo during CPR and primarily show that while adrenaline seems to favor the return of spontaneous circulation (ROSC) and survival to hospital discharge, it does not improve favorable neurological outcomes [6,7].

Coronary perfusion pressure (CPP) is predictive of myocardial blood flow. In the 1980s, it was believed that a threshold of 15–20 mmHg was necessary to achieve ROSC [8,9,10]. A more recent study has shown that the threshold value might be higher, around 35–40 mmHg [11]. Through its alpha-adrenergic effect, adrenaline improves myocardial flow, coronary perfusion, and cerebral blood flow [12,13,14]. 

End-tidal CO_2_ (EtCO_2_) is a key determinant and monitoring tool during CPR and is recommended for assessing prognosis and airway management [4,15]. EtCO_2_ also correlates with circulation and increases during CPR, along with cardiac index and CPP, and could be used to evaluate blood flow [16,17].

CPP is a relevant parameter for predicting ROSC, but it does not reflect cerebral and peripheral tissue perfusion. NIRS (near-infrared spectroscopy), a marker of regional oxygen saturation, has already been described in a pig model of cardiac arrest as an indirect tool to measure cerebral perfusion [18]. Cerebral NIRS might be useful for detecting ROSC and assessing the quality of chest compressions during CPR [19,20].

The aim of our study was to compare the increase in CPP with reduced adrenaline doses (0.5 and 0.25 mg) vs. standard doses of adrenaline during CPR on a pig model.

## 2. Materials and Methods

### 2.1. Ethical Statement

This study was approved by the Ethics Committee for Animal Experimentation of the Nancy University (APAFIS number 2019081910551467 V3). The procedure for the care and sacrifice of study animals was in accordance with the European Community Standards on the Care and Use of Laboratory Animals.

### 2.2. Surgical Preparation

Fifteen six-month-old male Landrace pigs were studied. They were acclimated for 7 days to reduce stress and were fasted overnight with free access to water in the university-affiliated animal laboratory. Just before the experiment, they were all pre-medicated with an intra-muscular injection of ketamine (15 mg·kg^−1^, Ketalar, Parke-Davis, Courbevoie, France) and midazolam (0.1 mg·kg^−1^, Hypnovel, Produits Roche, Neuilly sur Seine, France).

The experiment took place under general anesthesia induced by an intravenous bolus of propofol (1 mg·kg^−1^, propofol-lipuro 1%, B. Braun, Melsungen AG, Melsungen, Germany) through the right auricular vein. Animals were intubated (TeleflexIsis 7.5 I.D. mm, Teleflex Medical, Athlone, Ireland) and mechanically ventilated (Monnal T60, Air Liquide, Antony, France) in assisted-controlled mode (21% oxygen, tidal volume 10 mL·kg^−1^, respiratory rate of 15). Anesthesia was maintained with continuous infusion of sufentanil (0.2 μg·kg^−1^.min^−1^, Sufentanil, Mylan, Canonsburg, PA, USA), propofol (7 mg·kg^−1^.h^−1^, propofol-lipuro 2%, B. Braun Melsungen AG, Germany), and cisatracurium (0.9 mg·kg^−1^.h^−1^, Nimbex, GlaxoSmithKline, Brentford, Middlesex, UK). Saline infusion was administered during the preparation phase to maintain normovolemia (10 mL·kg^−1^.h^−1^). No other infusion was used, especially no buffering infusion. The temperature was also controlled during the experiment with an intrarectal thermometer.

Animals were monitored by a five-lead electrocardiogram, SpO_2_, and EtCO_2_ using the Monnal system (Irma CO_2_ probe Monnal, Masimo Corporation, Irvine, CA, USA).

Neck and femoral vessels were then dissected to insert a percutaneous introducer in the right internal jugular vein and the right femoral artery (Percutaneous sheath introducer Arrow^®^, Teleflex, Wayne, PA, USA). A pigtail catheter with a pressure sensor (5F Science Coactive pigtail tip pressure catheter, Transonic System, New York, NY, USA) was inserted in the right jugular vein down to the right atrium. The position of the probe was controlled by the pressure waveform. A pressure sensor (Millard^®^) was also inserted in the femoral artery up to the descending thoracic aorta. A transit time flow probe (Transonic Systems Inc., New York, NY, USA) was inserted around the right carotid artery to measure carotid blood flow. The cerebral NIRS sensors had two probes and were fixed on the left and right forehead region after shaving the head (Masimo SET O3 Sensor, Masimo Corporation, Irvine, CA, USA). The peripheric NIRS sensor was fixed on the anterior left leg after shaving (Inspectra StO_2_ sensor thenar Model 1615, Hutchinson Technology Inc., Hutchinson, MN, USA). The head remained in a standard horizontal supine position during the protocol. When all catheters were in place, a bolus of 10 UI.kg^−1^ of unfractionated heparine was administered through the jugular catheter to avoid clotting (Heparine Sodique Choay, Sanofi-Aventis, Paris, France). All models benefited from a 30-min pause for baseline measurements. Blood gases were controlled before intervention to check pH and pCO_2_ levels, and ventilatory parameters were modified if necessary to obtain a pH between 7.35 and 7.45. Mean arterial pressure during baseline recording had to be over 65 mmHg to start the experiment. If needed, a saline perfusion was administered to achieve the required pressure.

Arterial blood gas and hemoglobin levels were assessed in an acid–base and co-oxymeter analyzer (VetStat™, IDEXX Laboratories, Hoofddorp, The Netherlands). Lactate concentrations were determined using a Statstrip Lactate Xpress Meter (Nova Biomedical, Flintshire, UK). The animals’ heart and respiratory rates, as well as movements, were monitored. Any noticeable changes were tracked, and anesthesia was increased if necessary.

### 2.3. Experimental Protocol

**Figure 1:**
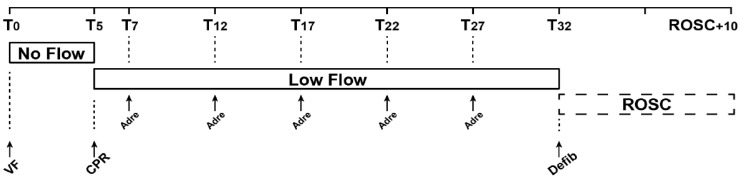
Experimental timeline. VF, ventricular fibrillation; CPR, cardio-pulmonary resuscitation; Adre, adrenaline injection; Defib, defibrillation.

The experimental protocol and timeline are presented in Figure 1. A simple randomization was carried out using a computer, and animals were randomly assigned to three groups of five animals each before the start of the experiment: 0.25 mg, 0.5 mg, or 1 mg of adrenaline. There was no blinding.

After surgical preparation of the model, the mechanical compression device (LUCAS^TM^ Physio-control, Lund, Sweden) was placed under the animal. This device was used to standardize the compression’s quality. The specimen was placed in a supine position. The device’s position was controlled and drawn on the animal’s chest to control the position during the experiment. The specimen was then secured by strapping the animal to the surgical table. A 30-min rest was then observed before starting the experiment.

Ventricular fibrillation was induced by three 9V batteries delivering a direct current via a pacing wire inserted in the right ventricle through the percutaneous introducer placed in the right internal jugular vein. T0 time was defined as blood pressure dropping below 40 mmHg. Ventilation was stopped, and five minutes of no-flow was observed.

Compressions were started at a rate of 100 per minute, and ventilation was resumed according to the European Resuscitation Council recommendations (100% oxygen, rate of 10/min, tidal volume of 10 mL·kg^−1^, PEEP 0) at T + 5 min [21].

After two minutes of compression, the first bolus of adrenaline (Adrenaline, Aguettant, Lyon, France) was administered and flushed with 10 mL of saline solution. Adrenaline was administered every 5 min. Each bolus in each randomized group was dissolved to inject the same volume of 1 mL and flushed with 10 mL of saline solution through the catheter placed in the right jugular vein. 

Aortic pressure, right atrial pressure, and carotid blood flow were monitored continuously.

In a cardiac arrest model, CPP can be estimated by measuring aortic–diastolic pressure (AoD) and right atrial diastolic pressure (RAD) [22,23].

After 32 min of resuscitation, pigs were defibrillated with a 200 J biphasic shock (TEC 8342 K, Nihon Khoden, Rosbach vor der Hohe, Germany). Up to five shocks were administered. If ROSC was achieved, 10 min of data were recorded. The animals were then sacrificed by injection of phenobarbital (0.1 mg·kg^−1^ of Exagon^®^, Axience, Pantin, France) (Figure 1). 

### 2.4. Objectives and Endpoints

#### 2.4.1. Main Objective

To compare the coronary perfusion pressure increase with reduced doses of adrenaline. Adrenaline reached its peak plasma concentration after 90 s [13].

#### 2.4.2. Secondary Objectives

To compare ROSC, the level of cerebral and peripheral tissue perfusion with reduced doses of adrenaline.

#### 2.4.3. Primary Endpoint

Increase in CPP with reduced doses of adrenaline compared with the CPP obtained with the recommended dose of 1 mg.

#### 2.4.4. Secondary Endpoints

ROSC rate;

Cerebral NIRS value 32 min after cardiac arrest according to adrenaline doses;

Tissue NIRS value 32 min after cardiac arrest according to adrenaline doses;

Cerebral NIRS value 10 min after ROSC according to adrenaline doses;

Tissue NIRS value 10 min after ROCS according to adrenaline doses;

EtCO_2_ levels according to adrenaline group.

### 2.5. Statistical Analysis

All hemodynamic data were recorded by IOX software at a frequency of 100 Hz. NIRS, EtCO2, blood gas, and lactate were recorded as described by the protocol.

All analyses were performed using R software (The R Foundation for Statistical Computing). The two-tailed significance level was set at *p* < 0.05.

Continuous variables are described using median (min, max) and categorical variables as counts (%). Continuous variables were compared using the exact Wilcoxon test or exact Kruskal–Wallis test, and categorical variables using Fisher’s exact test.

## 3. Results

Overall, 15 pigs were put in cardiac arrest by inducing ventricular fibrillation. No animals were excluded from the analysis, but there were some missing data. One of the two cerebral NIRS probes failed to provide data during the whole experiment for two pigs. In addition, during the experiment, there were technical issues with our pressure sensor inserted into the right atrium of the twelfth subject, and some data are missing (outlier data). These missing data were not analyzed.

Five animals were randomized in each group: 1 mg, 0.5 mg, and 0.25 mg.

Animals weighed between 45.4 and 66.3 kg. There was no difference between the three groups regarding basal parameters. ROSC occurred at the same frequency in each group (Table 1). In each group, we observed one death (20%)

CPP at T7 (before the first adrenaline injection) increased from 24.5 mmHg (12; 29.8) to 28.9 (21.2; 35.4) in the 0.25 mg group after 90 s (T8.5). The absolute difference between T7 and T8.5 was 4.93 (3.31; 15.2). In the 0.5 mg group, CPP rose from 28.6 mmHg (18.6; 33.5) to 39.6 (32.7; 52.5) with an absolute difference between T7 and T8.5 of 13.6 (11; 19). Finally, in the 1 mg group, CPP increased from 28.6 (18.6; 33.5) at T7 to 28.6 (18.6; 33.5) at T8.5 with an absolute difference of 18.4 (13.4; 30.4). There was no statistical difference between the three groups at T7, whereas, at T8.5, CPP was significantly higher in the 1 mg group, as well as the absolute difference when compared with 0.25 mg (*p* = 0.08 and *p* = 0.032). The difference remained non-statistically different between the 0.5 mg group and the 1 mg group (*p* = 0.056 and *p* = 0.22).

For the second and third injections, CPP after injection was significantly lower in the 0.25 mg group. At T13.5, CPP was 22.6 (13; 36.7) in the 0.25 mg group and 48.3 (30.2; 64.1) in the 1 mg group (*p* = 0.032). At T18.5, CPP was 24.1 (19.1; 28.3) in the 0.25 mg group and 38.2 (24.2; 69.9) in the 1 mg group (*p* = 0.032). 

**Figure 2:**
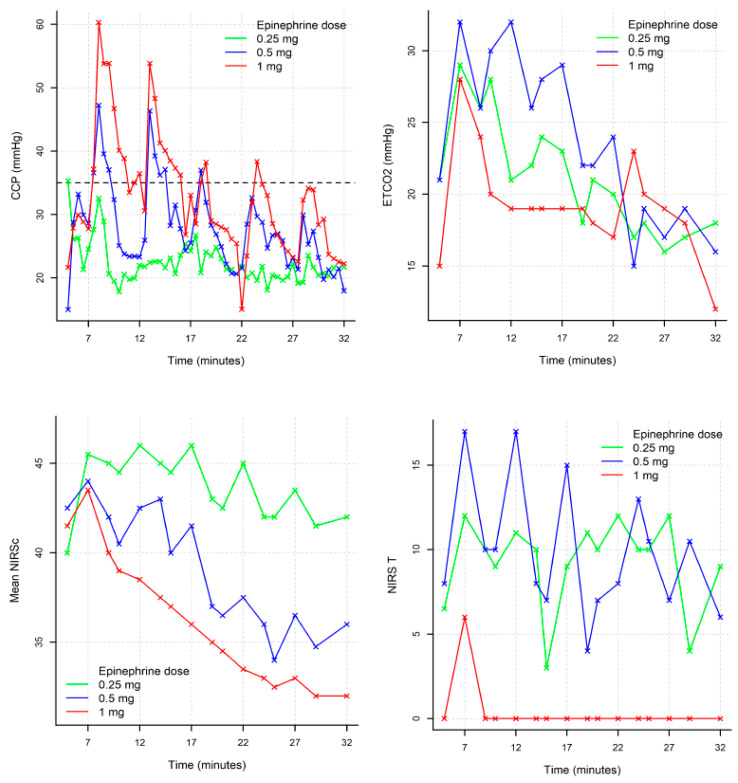
Variation in CPP, EtCO_2_, cerebral, and tissular NIRS for each group (median).

Additionally, the absolute difference between T22 and T23.5 (fourth injection) between the 0.25 mg group and the 1 mg was significant (*p* = 0.032). There was no statistical difference with the 0.5 mg group (Table 2 and Figure 2).

**Figure 3:**
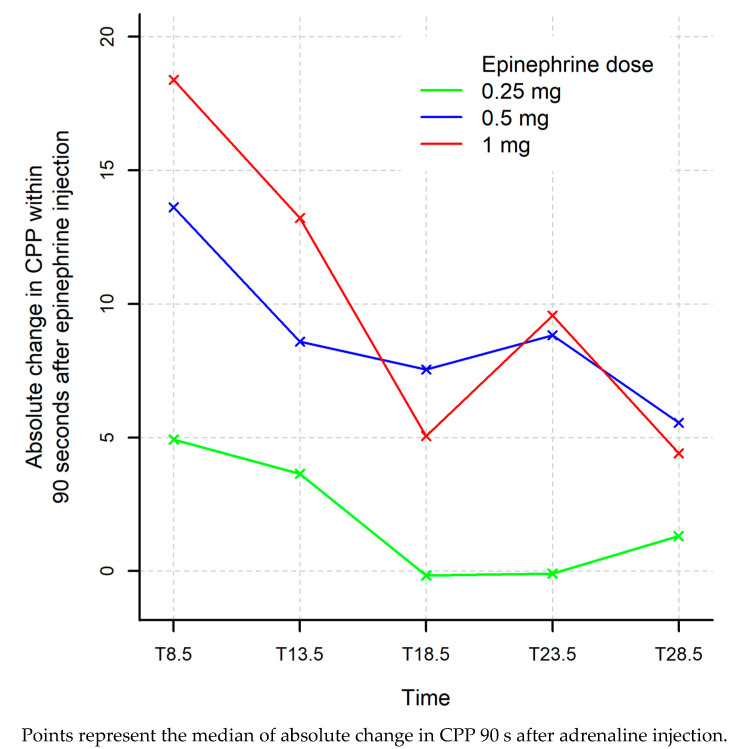
Absolute variation in CCP, 90 s after adrenaline injection in each group.

The threshold of 35 mmHg was reached 90 s after the first adrenaline injection in 20% of the 0.25 mg group, 60% of the 0.5 mg group, and 100% of the 1 mg group (*p* = 0.066; 0.25 vs. 1 mg *p* = 0.048; 0.5 vs. 1 mg *p* = 0.44). For the following injection, there was no statistical difference (Table 3 and Figure 3).

EtCO_2_ levels between T7 and T9 (two minutes after the first adrenaline injection) were not significantly different. EtCO_2_ is higher in the 0.5 mg group than the 1 mg group at T12, T14, and T17 with 32 (23; 35), 26 (20; 34), and 29 (22; 37), respectively (*p* = 0.016, *p* = 0.032, and *p* = 0.016). There was no significant difference for any other timing (Table 4) (Figure 2).

At T32, NIRSc was of 42% (39.5; 59.5) for 0.25 mg; 36% (32; 49) for 0.5 mg, and 32% (29.5; 43.5) for 1 mg (*p* = NS).

There was no statistical difference between the three groups after ROSC (Table 2) (Figure 2).

At T32, NIRSt was 9% (0; 12), 6% (0; 22), and 0% (0; 0) (*p* = 0.030; 0.25 mg vs. 1 mg *p* = 0.048 and 0.5 mg vs. 1 mg *p* = 0.048). Tissue NIRS were not statically different between the three groups after 10 min of ROSC (Table 2) (Figure 2).

## 4. Discussion

This study has shown that a 0.5 mg adrenaline bolus increases CPP after 90 s to the same level (with no statistical difference) as the standard dose of 1 mg in a pig cardiac arrest model, using mechanical compression. In comparison, 0.25 mg seems insufficient to increase CPP to the same levels as 1 mg (Figure 3).

### 4.1. Coronary Perfusion Pressure and Return of Spontaneous Circulation

CPP is probably the main determinant to achieve ROSC, and it has been demonstrated that higher CPP is associated with better chances of ROSC [8,9,11]. The threshold value of CPP during CPR remains unknown. In the 1990s, Paradis et al. proposed 15 mmHg, but a more recent study raised the value to 35–40 mmHg [8,11]. Because that value remains uncertain, it was assumed that the recommendation of 1 mg of adrenaline was sufficient to achieve ROSC [24], and the goal was to determine if reduced adrenaline doses could reach the same CPP levels as 1 mg. We noticed that the first bolus is the most effective and that the effect of adrenaline fades with time during CPR, especially with higher doses such as 1 mg. One explanation could be that receptors are more rapidly saturated with adrenaline at 1 mg. Hardig et al. showed that the first injection led to a higher peak in CPP in a pig model of cardiac arrest with repeated adrenaline doses. They also documented decreased EtCO_2_ level, cerebral tissue oximetry, and SpO_2_ after each injection, illustrating a decreased organ and brain perfusion [25]. Moreover, the CPP peak might not always imply better oxygenation. Wagner et al. showed that although CPP increases after adrenaline injection, continuous coronary artery flow average peak velocity increased only after the first bolus despite an increase in CPP during the following injections. The authors explained these results by a probable increase in local vascular resistance, meaning less oxygen for the myocardium [26].

In light of these results, it can be assumed that using 0.5 mg instead of 1 mg should be equally effective in achieving ROSC, especially since increasing to higher levels might not be useful because local vascular resistance might also increase.

Adrenaline used during CPR also causes post-resuscitation myocardial dysfunction by increasing myocardial oxygen consumption and favoring arrhythmia [27,28,29]. A balance is needed between decreasing the dose of adrenaline to protect myocardial function and sufficiently increasing the aortic pressure and CPP to obtain ROSC.

### 4.2. EtCO_2_ and CPP Interaction

EtCO_2_ values remained the highest in the 0.5 mg group throughout the experiment. EtCO_2_ is an essential, easy-to-use clinical monitoring tool during CPR. Monitoring EtCO_2_ is recommended to confirm endotracheal intubation, detect ROSC, and assess the quality of chest compressions [4,15,30,31]. As Sanders et al. showed in the 1980s, EtCO_2_ is correlated to CPP [17]. This relationship might disappear after an adrenaline injection. Adrenaline induces vasoconstriction of pulmonary vessels with an increased shunt effect and less CO_2_ elimination, explaining the decrease in the EtCO_2_ level after adrenaline injection [32,33,34]. This decrease in EtCO_2_ levels was also described in Hardig’s experiment with repeated injections of adrenaline [25]. In this experiment, although CPP was higher in the 1 mg group, EtCO_2_ values were statistically lower than in the 0.5 mg group. This could be explained by a potentially greater pulmonary shunt with 1 mg than 0.5 mg. However, EtCO_2_ values for 0.25 mg were not statistically different from the 1 mg group, probably because the increase in CPP was lower. 

### 4.3. Cerebral/Peripheral Organ Perfusion and Reduced Doses of Adrenaline

In our study, cerebral NIRS seemed to be higher after 32 min of CPR with reduced doses of adrenaline: 32% for 1 mg, 36% for 0.5 mg, and 42% for 0.25 mg (*p* = NS).

Cerebral NIRS reflects cerebral oxygenation and is widely used to monitor critically ill patients and during surgery [35,36,37,38]. Lately, it has been used more frequently during cardiac arrest and post-resuscitation care. Studies have shown that using a NIRS device in the field is feasible and that a higher regional oxygen saturation index (rSO_2_) during resuscitation is associated with ROSC and a higher chance of survival [20,39,40,41].

The adrenaline dose in our study significantly altered tissue NIRS. Higher adrenaline doses probably induced greater peripheral vasoconstriction resulting in a clear decrease in tissue NIRS values.

An interesting study on pigs from Reynolds et al. showed that tissue rSO_2_ decreased during CPR after administration of adrenaline. This decline is slightly more pronounced when receiving higher doses of adrenaline compared with standard doses, which supports the assumption of higher peripheral vasoconstriction [18].

It could be assumed that comparable results could be expected with cerebral rSO_2_, but Nosrati et al. showed in their study that there was no significant difference for measured cerebral NIRS between the group receiving placebo, adrenaline boluses, or adrenaline infusion [42]. The effects of adrenaline on cerebral blood flow and perfusion during CPR remain complex, especially because the effects of adrenaline differ when administered by infusion or boluses and at high or low doses [43,44]. Multiple studies have shown that it is likely that adrenaline causes neurological impairment by decreasing brain perfusion and cerebral microcirculation [25,34,45,46].

### 4.4. Limitations

This study has several limitations. First, it is based on a small population of only 15 specimens, and there were missing data for CPP for one specimen in the 0.25 mg group. There was no exclusion, but there were missing data for the cerebral NIRS probe that failed to provide data during the experiment for two pigs.

Although swine are the best experimental model to study cardiac arrest, all results might not be generalized to humans [47].

Second, the 0.25 mg group showed a significantly lower CPP than the 1 mg group, but ROSC was obtained in four of the five specimens in all three groups, which was unexpected.

Third, NIRS is only a reflection of cerebral oxygenation; it is not a measurement of cerebral blood flow or perfusion. No data on cerebral perfusion are available in this study; implementing an intracranial pressure probe would have complicated the model and was therefore not included in the study.

Fourth, it did not seem relevant to study biomarkers such as NSE or S100 B, as the experiment only lasted 30 min, and it is very likely that variations would not have been significant over such a short period. Furthermore, keeping the specimens alive for hours or days after the experiment was not an option in our laboratory.

## 5. Conclusions

The increase in CPP levels after administering adrenaline boluses of 0.5 mg was not significantly different from the recommended 1 mg dose in our experimental model of cardiac arrest. Moreover, we had the same rate of ROSC in both groups. Comparisons with data from cardiac arrest registries are necessary. Results are promising, and future human studies are warranted to assess the effectiveness of reduced boluses of adrenaline.

## Figures and Tables

**Table 1:** Baseline characteristics of specimen according to randomization group.

Baseline Characteristics	1 mg Adrenaline (*n* = 5)	0.50 mg Adrenaline (*n* = 5)	0.25 mg Adrenaline (*n* = 5)
Weight (kg)	61.1 (46.4; 65.7)	53.9 (50; 63.5)	55 (45.4; 66.3)
ETCO_2_ (mmHg)	44 (37; 51)	44 (41; 48)	47 (34; 55)
SpO_2_ (%)	99 (95; 100)	97 (93; 97)	96 (94; 97)
HR (/min)	93 (81; 106)	94 (81; 118)	101 (83; 126)
Temperature (Celsius)	38.8 (38.2; 40.8)	39.3 (38.9; 40.6)	39.4 (39.1; 39.6)
pH	7.39 (7.35; 7.49)	7.42 (7.38; 7.43)	7.38 (7.31; 7.47)
pO_2_ (mmHg)	102 (96; 143)	119 (99; 160)	96 (88; 194)
pCO_2_ (mmHg)	49 (42; 53)	49 (47; 51)	52 (40; 60)
Lactate (mmol/L)	1.7 (1.2; 2.4)	1.4 (1; 1.8)	1 (0.75; 2.3)
AP systolic (mmHg)	109 (82; 140)	108 (100; 114)	109 (89.5; 141)
RAP systolic (mmHg)	7.7 (3.12; 9.28)	7.18 (2.32; 8.88)	8.3 (4.79; 15)
AP diastolic (mmHg)	81.1 (58.4; 104)	74.1 (61.5; 83.7)	83.4 (52.1; 104)
RAP diastolic (mmHg)	3.83 (−1.49; 4.18)	3.06 (2.25; 5.17)	3.66 (0.902; 9.94)
CPP (mmHg)	76.9 (54.4; 106)	71 (58.5; 80.4)	79.8 (51.2; 100)
NIRSc Baseline (%)	54 (47; 59)	54.5 (46.5; 65.5)	57.5 (53.5; 69.5)
NIRSt Baseline (%)	41 (38; 62)	31 (20; 70)	39 (25; 43)
ROSC			
No	1 (20.0 %)	1 (20.0 %)	1 (20.0 %)
Yes	4 (80.0 %)	4 (80.0 %)	4 (80.0 %)

Values are median (minimum, maximum) for continuous variables, number (%) for categorical variables. Continuous variables were compared by exact Kruskal–Wallis test and categorical variables by Fisher’s exact test. HR, heart rate, AP, aortic pressure, RAP, right atrial pressure; CPP, coronary perfusion pressure; ROSC, return of spontaneous circulation.

**Table 2:** Variation in CPP before and 90 s after injection for each randomization group, cerebral and tissular NIRS results.

Parameter Measured	1 mg Adrenaline (*n* = 5)	0.50 mg Adrenaline (*n* = 5)	0.25 mg Adrenaline (*n* = 5)	*p*-Value 0.50 mg vs. 1 mg *	*p*-Value 0.25 mg vs. 1 mg *	*p*-Value Overall **
CPP						
T7	27.8 (24.3; 42.2)	28.6 (18.6; 33.5)	24.5 (12; 29.8)	0.55	0.15	0.25
T8.5	53.8 (37.8; 58.2)	39.6 (32.7; 52.5)	28.9 (21.2; 35.4)	0.056	0.008 ^‡^	0.0007 ^‡^
Absolute Δ T7–T8.5	18.4 (13.4; 30.4)	13.6 (11; 19)	4.93 (3.31; 15.2)	0.22	0.032 ^‡^	0.030 ^‡^
T12	36.5 (15.8; 40.4)	23.3 (12.1; 42)	22 (9.25; 29.6)	0.69	0.095	0.25
T13.5	48.3 (30.2; 64.1)	39.2 (23.5; 49.4)	22.6 (13; 36.7)	0.31	0.032 ^‡^	0.026 ^‡^
Absolute Δ T12–T13.5	13.2 (−6.28; 32.5)	8.59 (5.03; 22.1)	3.64 (−0.06; 14.7)	0.55	0.31	0.19
T17	33 (8.14; 59.7)	25.5 (19.5; 38.1)	24.3 (16.3; 51.8)	1.00	0.69	0.86
T18.5	38.2 (24.2; 69.6)	31.9 (27; 55.6)	24.1 (19.1; 28.3)	1.00	0.032 ^‡^	0.029 ^‡^
Absolute Δ T17–T18.5	5.05 (−3.2; 30.1)	7.54 (4.99; 17.5)	−0.159 (−0.342; 7.73)	0.69	0.41	0.27
T22	15.1 (5.38; 28.8)	21.6 (20.2; 33.8)	21.9 (6.01; 24.7)	0.22	0.73	0.36
T23.5	38.3 (20.3; 66.8)	29.7 (26.8; 45.1)	19.6 (10.5; 25.6)	1.00	0.063	0.028 ^‡^
Absolute Δ T22–T23.5	9.56 (3.22; 61.4)	8.82 (5.86; 11.3)	−0.0938 (−3.38; 4.45)	0.84	0.032 ^‡^	0.017 ^‡^
T27	23.3 (9.56; 57.7)	23.2 (18.9; 31.3)	22.2 (6.59; 31.3)	0.84	0.69	0.72
T28.5	34.1 (10.9; 62.1)	25.3 (21.8; 45.1)	23.5 (5.41; 48.7)	0.55	0.42	0.55
Absolute Δ T27–T28.5	4.41 (−0.577; 26.3)	5.55 (−1.39; 13.8)	1.31 (−1.18; 17.4)	1.00	0.55	0.86
ROSC10	52.5 (44.6; 58.4)	43.3 (3.59; 66)	54.9 (25.6; 86.1)	0.86	0.86	0.83
NIRSc						
Baseline	54 (47; 59)	54.5 (46.5; 65.5)	57.5 (53.5; 69.5	1.00	0.29	0.36
T32	32 (29.5; 43.5)	36 (32; 49)	42 (39.5; 59.5)	0.46	0.39	0.34
ROSC10	51.8 (42.5; 58.5)	47 (42; 50)	46.8 (46; 49)	0.69	1.00	0.92
NIRS T						
Baseline	41 (38; 62)	31 (20; 70)	39 (25; 43)	0.38	0.52	0.64
T32	0 (0; 0)	6 (0; 22)	9 (0; 12)	0.048 ^‡^	0.048 ^‡^	0.030 ^‡^
ROSC10	30 (23; 37)	38.5 (28; 57)	29 (0; 43)	0.23	1.00	0.47

CPP, coronary perfusion pressure; Absolute Δ, absolute difference; NIRS, near-infrared spectroscopy; ROSC, return of spontaneous circulation. Values are median (minimum, maximum). ^‡^ *p* is significant < 0.05 * *p*-value from Wilcoxon test. ** *p*-value from exact Kruskal–Wallis test.

**Table 3:** CPP threshold of 35 mmHg 90 s after adrenaline injection in the three compared groups.

CPP >35 mmHg	1 mg Adrenaline (*n* = 5)		0.50 mg Adrenaline (*n* = 5)		0.25 mg Adrenaline (*n* = 5)		*p*-Value 0.50 mg vs. 1 mg	*p*-Value 0.25 mg vs. 1 mg	*p*-Value * Overall
	N	n (%)	N	n (%)	N	n (%)			
T8.5	5	5 (100 %)	5	3 (60 %)	5	1 (20 %)	0.44	0.048 ^‡^	0.066 ^‡^
T13.5	5	4 (80 %)	5	3 (60 %)	5	1 (20 %)	1.00	0.21	0.30
T18.5	5	3 (60 %)	5	2 (40 %)	4	0 (0 %)	1.00	0.17	0.30
T23.5	5	3 (60 %)	5	2 (40 %)	4	0 (0 %)	1.00	0.17	0.30
T28.5	5	2 (40 %)	5	1 (20 %)	5	1 (20 %)	1.00	1.00	1.00
ROSC10	3	3 (100 %)	4	2 (50 %)	4	3 (75 %)	0.43	1.00	0.71

N, number of data available; CPP, coronary perfusion pressure. ^‡^ *p* of significant value <0.05 * *p*-value from Fisher’s exact test.

**Table 4:** EtCO_2_ variations before and after adrenaline injection.

ETCO_2_ (mmHg)	1 mg Adrenaline (*n* = 5)		0.50 mg Adrenaline (*n* = 5)		0.25 mg Adrenaline (*n* = 5)		*p*-Value 0.50 mg vs. 1 mg *	*p*-Value 0.25 mg vs. 1 mg *	*p*-Value Overall **
	n	Median (Min–Max)	n	Median (Min–Max)	n	Median (Min–Max)			
T7	5	28 (23; 36)	5	32 (23; 37)	5	29 (9; 40)	0.71	0.88	0.79
T9	5	24 (18; 31)	5	26 (17; 30)	5	26 (16; 28)	0.84	0.73	0.91
Absolute Δ T7–T9	5	−5 (−11; −2)	5	−6 (−9; −4)	5	−3 (−13; 7)	1.00	0.55	0.59
T12	5	19 (16; 26)	5	32 (23; 35)	5	21 (16; 34)	0.016 ^‡^	0.45	0.032 ^‡^
T14	5	19 (12; 22)	5	26 (20; 34)	5	22 (18; 27)	0.032 ^‡^	0.21	0.052
Absolute Δ T12–T14	5	0 (−6; 1)	5	−3 (−9; −1)	5	0 (−8; 3)	0.29	0.75	0.30
T17	5	19 (15; 23)	5	29 (22; 37)	5	23 (12; 34)	0.016 ^‡^	0.48	0.049 ^‡^
T19	5	19 (16; 24)	5	22 (15; 31)	5	18 (10; 27)	0.69	1.00	0.84
Absolute Δ T17–T19	5	0 (−3; 9)	5	−7 (−10; −5)	5	−3 (−7; 0)	0.008 ^‡^	0.17	0.005 ^‡^
T22	5	17 (14; 23)	5	24 (13; 29)	5	20 (7; 33)	0.29	0.61	0.49
T24	5	23 (12; 26)	5	15 (12; 29)	5	17 (8; 29)	0.72	0.84	1.00
Absolute Δ T22–T24	5	0 (−2; 7)	5	−2 (−9; 0)	5	−4 (−5; 1)	0.13	0.071	0.11
T27	5	19 (11; 29)	5	17 (10; 32)	5	16 (7; 34)	0.89	0.81	0.95
T29	5	18 (11; 28)	4	19 (14; 26)	5	17 (6; 20)	0.78	0.41	0.66
Absolute Δ T27–T29	5	0 (−1; 2)	4	−2 (−6; 0)	5	−1 (−14; 2)	0.19	0.49	0.35

N, number of data available; Absolute Δ, absolute difference ^‡^ *p* of significant value < 0.05 * *p*-value from exact Wilcoxon test. ** *p*-value from exact Kruskal–Wallis test.

## Data Availability

The datasets used and/or analyzed during the current study are available from the corresponding.
